# Investigation of neomycin biodegradation conditions using ericoid mycorrhizal and white rot fungal species

**DOI:** 10.1186/s12896-022-00759-1

**Published:** 2022-10-11

**Authors:** Åke Stenholm, Mikael Hedeland, Curt E. Pettersson

**Affiliations:** Department of Medicinal Chemistry, Analytical Pharmaceutical Chemistry, Uppsala University, BMC Box 574, 751 23 Uppsala, Sweden

**Keywords:** Neomycin, Biodegradation, UHPLC-Q-TOF MS, *Trametes versicolor*, *Rhizoscyphus ericae*

## Abstract

**Background:**

In the search for methods to biodegrade recalcitrant compounds, the use of saprotrophic fungi and white rot fungi, in particular belonging to the phylum Basidiomycota, has gained interest. This group of fungi possesses a battery of unspecific extracellular enzymes that can be utilized in the biodegradation of preferably phenolic compounds. In this work, it was investigated under which conditions the white rot fungus *Trametes versicolor* and the ericoid mycorrhizal fungus *Rhizoscyphus ericae* (belonging to the phylum Ascomycota) could be used to biodegrade the antibiotic aminoglycoside neomycin at co-metabolic conditions in which external nutrients were supplied. Furthermore, it was also investigated whether a biodegradation could be accomplished using neomycin as the sole nutrient.

**Results:**

The results show that both species can biodegrade neomycin 70% under co-metabolic conditions during a one-week time course and that *Rhizoscyphus ericae* is able to use neomycin as sole nutrient and to approximatively biodegrade it 60% under chosen non co-metabolic conditions.

At selected conditions, the biodegradation of neomycin using *Rhizoscyphus ericae* was monitored by oxidation products of D-ribose which is a hydrolysis product of neomycin.

**Conclusion:**

The results are of general interest in the search for fungal species that can biodegrade recalcitrant compounds without the need of external nutrients. The key future application area that will be investigated is purification of waste from recombinant protein production in which neomycin, nutrients and *E. coli* with neomycin resistance genes are present.

**Supplementary Information:**

The online version contains supplementary material available at 10.1186/s12896-022-00759-1.

## Introduction

**Fig. 1:**
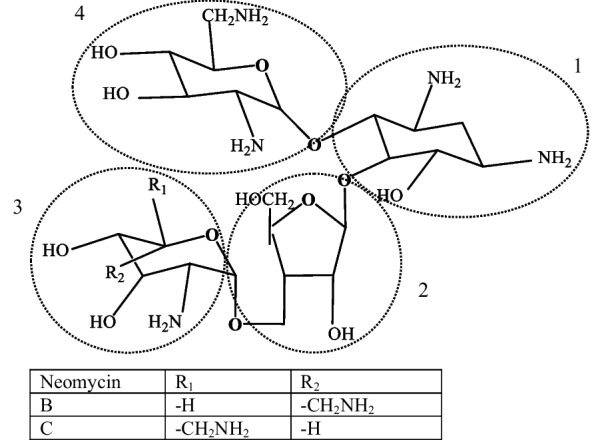
Chemical structures of neomycin B and C. 1: 2-DOS, 2: D-ribose, 3: neosamine B/C, 4: neosamine C

Neomycin is an aminoglycoside which is used in human and in veterinary medicine [1]. In Fig. 1, the chemical structures of neomycin B and C are shown. Neomycin is a mixture of neomycin B and neomycin C. The latter component is present in approximately 5–15% of the mixture. Neomycin A, which is an inactive degradation product of neomycin B and C, is present at concentrations < 1% [2]. Neomycin A (neamine) is composed of D-neosamine (neosamine C) and 2-deoxystreptamine (2-DOS). Neomycin B is composed of four parts: D-neosamine, L-neosamine (neosamine B), 2-DOS and D-ribose. Neomycin C contains the same components, except that two D-neosamine moieties are present. Neobiosamine B and C are formed by 2 and 3 in Fig. 1 dependent on the initial isomers.

Neomycin was discovered 1949 [3] and is active against both Gram-negative and Gram-positive bacteria. Neomycin targets functional centers within the bacterial ribosome. Interestingly, it has been shown that neomycin cannot only target the bacterial, but also the eukaryotic ribosome in the common yeast species *Saccharomyces cerevisiae* [4]. However, the vast majority of fungi, are not affected by aminoglycosides [5]. Aminoglycoside antibiotics are mostly excreted unmetabolized and are therefore present in wastewaters [6, 7]. They are recalcitrant [8], and can therefore contribute to the build-up of antibiotic resistance. The increased concentration levels of antibiotics like neomycin in wastewater streams leading to wastewater treatment plants (WWTPs) thus constitutes a breeding ground for antibiotic resistant bacteria [9]. Examples of bacteria that are resistant to neomycin include *Streptococcus pyogenes* [10], *Escherichia coli* [11] and *Staphylococcus aureus* [12].

The presence of residual neomycin in the natural environment is scarcely investigated. One reason is that the neomycin molecule is difficult to analyze since it is positively charged in rivers, lakes and WWTPs. Furthermore, it does not contain chromophores. In one study, LC MS/MS was used to quantify neomycin in raw and WWTP-treated wastewaters in Tunisia [8]. In another investigation, the same analysis technique was used to analyze water supply systems in food-producing animal farms [13]. In the Tunisian study, the influent neomycin concentration was determined to 16.4 ng mL^−1^. In the animal farm study, the water contained 32 µg neomycin L^−1^.

Since neomycin is recalcitrant, it is to a lesser extent biodegradable in WWTPs. Abiotic removal processes except heat treatment can be excluded mainly because of the compound´s physico-chemical properties [14]. Biodegradation of neomycin by fungi can be an interesting alternative. Fungi are eukaryotes. To this group belong both single-celled and multi-celled fungi. The multi-celled fungi include smuts, mildews, molds and mushrooms. They have a complex cellular organisation which provides an overall advantage over unicellular eukaryotic organisms like yeasts and rusts (fungi) but also over procaryotic bacteria when it comes to removal of pollutants [15].

A white rot fungus species, *Trametes versicolor* (*T. versicolor*) belonging to the phylum Basidiomycota (mushroom), has recently been investigated for its ability to biodegrade neomycin in vitro and in vivo [14]. In the cited study, it was concluded that it is possible to remove neomycin up to 80% in a batchwise process using polyurethane foam (PUF) immobilized fungal mycelia (in vivo) and by using a co-metabolism strategy in which glucose and ammonium tartrate are the carbon and nitrogen sources of choice. The investigation was conducted under non-sterile conditions at pH 4.0 to suppress bacterial growth [16].

A specific future application area for fungal processes is purification of waste from molecular genetic processes with emphasis on recombinant protein production using *E. coli* [17]. In these processes, neomycin concentration levels up to 50 mg L^−1^ could be expected. In a batchwise WWTP upstream process, the presence of additionally added nutrients may be acceptable. However, a process in which neomycin can act as nutrient (sole carbon and nitrogen supply) is worth investigating because of cost and simplicity aspects.

Carbon and nitrogen are needed by fungi for growth. The carbon to nitrogen ratio (C:N) of a substrate influences the growth [18]. There are contradictory statements regarding the most suitable composition of a fungal nutrient for biodegradation purposes. Specifically, *T. versicolor* has been optimized to decolorize Kraft pulp mill using nutrients with C:N ratios up to 405 (w/w) [19]. On the other hand, it has been shown in microcosms that extra N-addition in the form of ammonium nitrate stimulated the general fungal growth at the expense of bacterial growth [18].

A fungal species which has been proven to be able to use recalcitrant nitrogen containing compounds as nutrients is the mushroom *Rhizoscyphus ericae* (*R. ericae*) [20]. It is an ericoid mycorrhizal symbiotic species which belongs to the phylum Ascomycota. This phylum contains parasitic, symbiotic and saprotrophic species. Their biodegradation capability is characterized by the involvement of intracellular cytochrome P450 enzymes [21]. *R. ericae* colonizes acidic and nutrient-poor environments in the arctic and alpine tundras, and has been extensively investigated for its ability to use complex substrates as nutrients [22, 23]. In a study from 1990, Leake and Read investigated how the growth of *R. ericae* mycelia was affected by various nitrogen sources, including ammonium, alanine and bovine serum albumin (BSA) [22]. The pH of the media was adjusted to 4.0. It was concluded that the mycelial growth was not affected by the choice of nitrogen source. Thus, it is a testimony that this fungus can assimilate nitrogen despite the great variation in molar mass of the different sources which ranged from 18 g mol^−1^ (ammonium) to 67,000 g mol^−1^ for BSA. More recently, the genome of *R.ericae* was sequenced [24]. The investigation confirmed the ability of *R. ericae* to digest recalcitrant compounds by the presence of genes coding for carbohydrate-active enzymes (CAZymes), lipases and proteases.

The general aim in the present study was to investigate under which nutritional conditions the fungal species *T. versicolor* and *R. ericae* can biodegrade neomycin. The research topic is novel and to our knowledge, this is the first time *R.ericae* is investigated whether it can be used to biodegrade pharmaceuticals. Furthermore, the biodegradation patterns and kinetics presented are not previously investigated.

## Materials and methods

### Chemicals, filters and flasks

Neomycin trisulfate hydrate (≥ 600 µg neomycin mg^−1^, dry basis) was purchased from Sigma Aldrich (Buchs, Switzerland). Ammonium tartrate dibasic (≥ 98%) and D-( +)-glucose (≥ 99.5%) from Sigma Aldrich and sulfuric acid (95–97%; Merck Millipore (Darmstadt, Germany) were used to prepare a nutrient solution at pH 4.0. Acetonitrile, Optima™, LC/MS grade from Fisher Scientific (Reinach, Switzerland), water for chromatography Lichrosolv® LC–MS grade (Merck Millipore) and formic acid (98–100%) from Merck Millipore were used in the UHPLC-Q-TOF analyses. Polyurethane foam (PUF) filter cartridges for aquariums (Pickup 200 EHEIM, Germany) were used for the immobilization of *T. versicolor*. The porosity of this open-cell filter type with a reticular structure is between 95 and 98%. Water, purified at Cytiva (Uppsala, Sweden) using a Milli-Q® water system (Milli-Q Millipore 0.22 µm serial no 1550, Massachusetts, USA) was used in the UHPLC-Q-TOF mobile phases, and tap water softened at Cytiva, Uppsala, Sweden was used in the experimental solutions that contained fungal cultures, thereby providing trace amounts of minerals. D-ribose with a purity of ≥ 99.0% from Merck, was used as UHPLC-Q-TOF standard.

Glass fibre prefilters, cat. no. AP 2,504,700 from Merck Millipore (Burlington, USA) were used to determine initial biomass weights of *T. versicolor* mycelia. Munktell, 1003, 9 cm-filters (Munktell Filter AB, Sweden) were used to determine the biomasses of *R. ericae* in experimental cultures. Furthermore, 300 µL fixed insert amber vials (Agilent Technologies, Santa Clara, USA) were used both for calibration standards and samples. For the biodegradation experiments, 500 mL Duran® Erlenmeyer flasks with bottom-baffles and air permeable lids from Sigma Aldrich were used.

### Fungi

*T.versicolor* (strain AG 1383) was donated from Culture Collection of Basidiomycetes (CCBAS, Institute of Microbiology, Academy of Sciences of the Czech Republic, Prague). *R. ericae* (*Hymenoscyphus ericae*) was donated by Dr. Martin Vohnik from Institute of Botany (Academy of Science of the Czech Republic, Prague). This strain is derived directly from the type culture (Acc. Number UAMH6735) in the collection at the University of Alberta, GenBank number AJ319078, originally isolated from a *Calluna Vulgaris* hair root in the UK. Mycelia of both strains were grown on malt agar and transported to Sweden at refrigerated conditions (2–8 °C).

### Instrumentation

An Edmund Bühler KS10 shaking table from Tübingen, Germany was used to stir the Erlenmeyer flasks (E-flasks). A Termaks, TS 9053 drying oven from Bergen, Norway was used to dry mycelia containing agar plugs.

An Agilent Technologies 6550 iFunnel Q-TOF LC/MS system (Agilent Technologies, Santa Clara, CA, USA), including an Agilent Technologies 1290 Infinity UHPLC system consisting of a 1260 iso pump (G1310B), 1290 binary pump (G4220A), thermostat (G1330B), 1290 sampler (G4226A), 1290 thermostated column compartment (G1316C), and electrospray ionization (ESI) system, was used for qualitative and quantitative analysis. Agilent MassHunter software (V. 06.00) was used for instrument control, data acquisition and processing. For separation purposes, a zwitterionic UHPLC column (SeQuant® ZIC®-cHILIC, 100 × 2.1 mm, 100 Å, 30 µm, Merck, Darmstadt, Germany) was used. Solid Phase Extraction (SPE) was performed with Isolute® C18 (EC) 100 mg 1 mL SPE columns, Biotage (Uppsala, Sweden). A pH meter, Mettler Toledo Steven Compact ID 12–137 (Greifensee, Switzerland) was used to adjust and measure the pH in all fungal experiment solutions.

### Experimental procedures

#### Preparation and composition of nutrient solutions

In the nutrient solution that was used for inoculation purposes, sulfuric acid was added to a 1000 mL measuring flask containing 6.0 g D-glucose, 3.3 g ammonium tartrate, and 900 mL softened tap water to obtain pH 4.0. The pH was selected with the aim of suppressing bacterial growth. The carbon-to-nitrogen ratio (C:N ratio) was 6.5. The carbon and nitrogen contents in a 300 mL volume of nutrient solution (experimental volume) were 971 and 150 mg respectively. The procedure was similar for nutrient solutions that contained alternative compositions of glucose and ammonium tartrate that were used in the biodegradation experiments (see section “Experimental design”).

#### Cultivation of* T. versicolor* and* R. ericae*

*T. versicolor* and *R. ericae* were grown on petri dishes with extracted malt agar from the Swedish National Veterinary Institute (Uppsala, Sweden). Growth was conducted in an aseptic environment where a malt agar plug was removed with a 10 mm (internal diameter) punch, previously sprayed with 70% EtOH and allowed to dry, from a prior culture of mycelia to a new dish. The petri dishes were kept under dark conditions by wrapping with aluminum foil for one week (*T. versicolor*) and two weeks (*R. ericae*) and then storing in a refrigerator at a temperature of + 4 °C. This sub-culturing procedure was performed every second month.

#### Inoculation and PUF immobilization of* T. versicolor*

PUF pieces, cut in centimeter sized cubes, with a total weight of approximately 2.7 g were added to nineteen baffled E-flasks each. These flasks were filled with 300 mL nutrient solution each and then autoclaved at 125 °C for 30 min using a Certoclav CV-EL 12 L autoclave (Leonding, Austria). The flasks were then cooled to ambient temperature. In an aseptic environment, fifteen malt agar plugs containing cultivated *T.versicolor* were added to each flask. For twelve days prior to the experiments, the flasks were kept in the dark, covered with aluminum foil, and mounted on a shaking table operating at a low shaking speed (50 min^−1^) at room temperature. After 12 days of inoculation/immobilization, two of the flasks (heat killed controls (HKCs)), were autoclaved once more. The HKCs were aimed for monitoring biosorption to dead *T.versicolor*. Immobililization is motivated by increased suppression of bacterial growth, increased secretion of extracellular enzymes and less residual mycelia in the liquid broth [25–27].

#### Inoculation of* R. ericae*

Previously non-published experiments, performed by the authors have shown that it is difficult to immobilize *R. ericae* on PUF. The mycelia did not colonize the PUF-pores, like the mycelia of *T. versicolor*. Therefore, the inoculation was performed in the absence of PUF. Twelve 300 mL baffled E-flasks were filled with 300 mL nutrient solution each, autoclaved and cooled according to the *T. versicolor* procedure. Twenty agar plugs containing cultivated *R. ericae* mycelia, were added to each flask. The same inoculation conditions (time, darkness and shake speed) as for *T.versicolor* were maintained. Two of the flasks (HKCs) were after 12 days autoclaved once more.

#### Biomass calculations

Punched pieces of the cultivated biomasses of *T.versicolor* and *R. ericae* were weighed after storage in a refrigerator for two weeks (see section “Cultivation of *T. versicolor* and *R. ericae”*). The weights of the initial biomasses (BM) were later used in the calculation of initial carbon biomass (C:BM) ratios. These ratios thus give information on the relation between carbon content in nutrients and neomycin, and BM at the start of the inoculation step.

The used methodology by which agar plugs containing fungal mycelia are dried for 20 min at 105 °C to determine initial biomasses is previously described [14]. In the present study, 15 agar plugs containing fungal mycelia from *T. versicolor* and 20 agar plugs containing *R. ericae,* were used for this purpose. The mean weight of the biomass in each *T.versicolor* and *R.ericae* plug was 2.6 mg ± 4.8% (*n* = 10) and 4.5 mg ± 6.7% (*n* = 10) respectively. Thus, the total biomass weight in 15 T*.versicolor* and 20 *R.ericae* agar plugs was 39 and 90 mg respectively.

In a separate study, BM of *R. ericae* was determined after (i) The 12:th day of the inoculation step (BMRe), and (ii) After 168 h biodegradation in six experimental cultures which did not contain any neomycin but only nutrient solution (NS0Re), 34 mg neomycin L^−1^ in the presence of nutrient solution (NS4Re), 34 mg neomycin L^−1^ (Neo1Re), 1.0 g neomycin L^−1^ (Neo2Re), 6.0 g neomycin L^−1^ (Neo3Re) and 15 g neomycin L^−1^ (Neo4Re). The four last experiments contained solely neomycin solutions. The elevated neomycin concentrations (> 34 mg L^−1^) were motivated by the desire to measure the biomass growth at the same conditions that were used in the biodegradation experiments. See Table 1 for experimental culture compositions of neomycin containing samples. Neomycin sulfate was easily dissolved at room temperature in water, even at a neomycin sulfate concentration of 22 g L^−1^ (15 g neomycin L^−1^). The experiments were performed in duplicate. The procedure was as follows: The filter that was going to be used to filtrate BM was initially dried in an oven for 20 min at 105 °C and allowed to cool to room temperature. Thereafter, BM was filtrated by gravity using the dried filter. BM was washed excessively with Milli-Q water to get rid of neomycin residues. Washed BM was dried for 20 min on the filter paper at 105 °C and allowed to cool to room temperature. Then the weight of the filterpaper and BM was determined, and finally, the previously determined weight of the filter paper was subtracted.

**Table 1:** Neomycin containing compositions that were used in the biodegradation experiments. Tv and Re are denotations of *T. versicolor* and *R. ericae*. HKC is heat control. NS and Neo are abbreviations of nutrient solution and neomycin

Experiment	Glucose (g L^−1^)	Ammonium tartrate (g L^−1^)	Neomycin (g L^−1^)	Initial biomass (g)
NS1Tv	0.060	0.045	0.034	0.039
NS2Tv	0.24	0.20	0.034	0.039
NS3Tv	1.2	0.90	0.034	0.039
NS4Tv	6.0	3.3	0.034	0.039
NSHKCTv	6.0	3.3	0.034	0.039
NS4Re	6.0	3.3	0.034	0.090
NSHKCRe	6.0	3.3	0.034	0.090
Neo1Tv	0.0	0.0	0.034	0.039
Neo1Re	0.0	0.0	0.034	0.090
Neo2Tv	0.0	0.0	1.0	0.039
Neo2Re	0.0	0.0	1.0	0.090
Neo3Tv	0.0	0.0	6.0	0.039
Neo3Re	0.0	0.0	6.0	0.090
Neo4Tv	0.0	0.0	15	0.039
NeoHKCTv	0.0	0.0	15	0.039
Neo4Re	0.0	0.0	15	0.090
NeoHKCRe	0.0	0.0	15	0.090

#### Experimental design

After the 12-day inoculation period, the broths were separated from mycelia immobilized PUF-pieces (*T. versicolor*) and the free mycelia (*R. ericae*) in most of the flasks, leaving solely the solids in the flasks. In the experiments with titles beginning with NS4, no decanting took place (see Table 1). In these experiments, neomycin was added to the inoculation solutions by gentle stirring. To the other flasks, differently composed solutions (300 mL each) were added to the flasks. The main purpose of the design was to evaluate to what extent neomycin could be biodegraded with and without the presence of other nutrients. The neomycin solutions that were added to the fungal cultures included softened tap water which was adjusted to pH 4.0 using 0.1 M sulfuric acid. These solutions were not autoclaved. In Table 1, the compositions are shown. Some of the experiments which were performed with *T. versicolor* and contained nutrient solution (NS) at different concentrations, were excluded for *R. ericae*. This was done mainly because this species had previously shown excellent capabilities to grow and survive on toxic nitrogen containing substances [20]. The flasks were then subjected to biodegradation experiments that lasted for 168 h. The flasks were covered with aluminum foil and agitated (50 rpm). Samples (300 µL) were pipetted at 0, 1, 2, 4, 6, 8, 24, 30, 48, 72, 96, 120, 144 and 168 h. They were immediately frozen at − 20 °C. Prior to UHPLC-Q-TOF MS analyses, the samples were thawed and worked-up with SPE. See section “Solid Phase Extraction”.

In Table 1, the experimental titles and conditions are shown. Each experiment was performed in duplicate except for NS4Tv that was done in triplicate and HKCs that were not replicated. The reason for the choice of the four HKC experimental conditions was that neomycin adsorption to dead mycelia may be affected (i) by the choice of species and (ii) by the neomycin and NS concentrations. The experiments that included neomycin concentrations > 34 mg neomycin L^−1^ excluding NS were motivated by the wish to understand whether NS can be used as sole nutrient. The NS composition in NS4TV and NS4Re approximately resembles the carbon and nitrogen concentration in the NS that is used in the recombinant protein production application.

The compositions in Table 1 were used to build a model matrix X aimed for Principle Component Analysis (PCA). In this matrix (see Additional file 1: Appendix 1), a number of independent variables were used. These were:

C_NS_ (amount of carbon in NS), N_NS_ (amount of nitrogen in NS), C_neo_ (amount of carbon in neomycin), N_neo_ (amount of nitrogen in neomycin), C_tot_ (total amount of carbon), N_tot_ (total amount of nitrogen), C_tot_:N_tot_, C_neo_:BM (initial biomass), C_tot_:BM (initial biomass). Two dependent variables were included in the PCA matrix: These were “Removal degree” and “Removal amount”.

#### Solid phase extraction

Thawed collected fractions from the biodegradation experiments were purified from lipids originating from the immobilized mycelia [28].

The SPE columns were first activated with 1.0 mL of acetonitrile and then equilibrated with 1.0 mL NS containing 6.0 and 3.3 glucose and ammonium tartrate L^−1^ respectively. The pH in NS was adjusted to 4.0 using sulfuric acid. Then, 300 µL collected samples were loaded onto the SPE-columns twice and the non-retarded neomycin was eluted with an applied pressure using a Pasteur pipette rubber bulb. The reason for the choice of NS as equilibration solution was that even if many of the experiments did not contain NS, it was considered to be important to have a consistency in the SPE procedure. The SPE recovery was investigated by preparing two QC samples, containing neomycin at concentrations of 10.0 and 34.0 mg L^−1^ (dissolved in NS (SPE equilibration solution)) followed by SPE and analyzing them by triplicate injections. The mean recovery was calculated to 94 and 92% respectively. See Eq. (1). The concentration was determined using external standards according to section “Quantitative analysis of neomycin”.

1 $$SPE\,recovery \left( \% \right) = \frac{Conc.\,determined\,in\,SPE\,eluates\,\times\,100}{{Initial\,QC\,sample\,conc.}}$$

#### Quantitative analysis of neomycin

The analyses were performed using external standards. The sample recovery for the SPE procedure was > 90% (see section “Solid Phase Extraction”). No matrix effects in SPE worked up samples that affected the neomycin quantification could be seen according to the initial neomycin concentration levels in the undiluted samples. They were all between 30 and 34 mg L^−1^ (see Figs. 4 and 6). The external standards consisted of neomycin dissolved in solely NS to 3.0, 6.0, 14.0, 20.0, 27.0 and 34.0 mg L^−1^. The NS concentration was similar with the inoculation and SPE equilibration solution, that is 6.0 and 3.3 g glucose and ammonium tartrate L^−1^ respectively at pH 4.0. Using the selected zwitterionic SeQuant ZIC-cHILIC column and the chosen instrumental settings, it was noticed that the composition of the neomycin solvent (different concentration levels of NS) and also pure MilliQ water did not alter the neomycin ionization degree or the linearity of the calibration curves. For experiments in which exceeded concentration levels of neomycin were used, dilutions of the collected fractions with MilliQ water were made in such a manner that the results would fit to the used external standards. For these samples, the SPE procedure (see section “Solid Phase Extraction”) was performed before the dilution step.

The zwitterionic SeQuant ZIC-cHILIC column was thermostated at 60 °C to decrease peak width and asymmetry. The choice of this temperature was based on initial experiments that were performed at 30, 45 and 60 °C. It was observed that by increasing the temperature, the neomycin retention times were shortened and the peak widths and asymmetries were decreased. The mobile phases consisted of A; acetonitrile: water (5:95 (v/v)) with 0.1% formic acid and B; acetonitrile:water (95:5 (v/v)) with 0.1% formic acid. Initially, 100% B was maintained isocratically for one minute. Thereafter, mobile phase B decreased linearly to 5% while mobile phase A increased to 95% during a time period of 1.5 min. This composition was held for 4.5 min. Thereafter, the column was equilibrated to 100% B during a time period of 0.5 min. This composition was held to the end of the run (18 min). The flow rate was 0.3 mL min^−1^ and the injection volume was 15 µL. The MS settings were: capillary voltage 3.5 kV, gas temperature 200 °C, sheath gas temperature 350 °C, sheath gas flow 11 L min^−1^ and a nozzle voltage of 1.0 kV. MS data were acquired in the *m/z* range of 100–1700. The ESI ionization was performed in positive mode and the instrument was tuned and calibrated every day prior to use. The external calibration standards containing neomycin in the 3.0 to 34 mg L^−1^ concentration range were co-analyzed together with SPE-worked up samples and pure nutrition solution (blanks) in each sample sequence. The proton neomycin adduct at *m/z* 615.3201 (exact mass) was used in the quantification. The integrated peaks include both abundant neomycin isomeric forms (B and C) since they are not separable using the chosen SeQuant® ZIC®-cHILIC column and gradient elution The reference mass ions which were used for internal mass calibration (lock masses) were *m/z* 121.0509 and 922.0098. Three QC samples with the concentrations 10.0, 25.0 and 30.0 mg L^−1^ were used for determining the accuracy. It was 86.2, 99.3 and 99.7% (*n* = 3), respectively. The linearity (*r*, i.e., the Pearson product-moment correlation coefficient) of 0.9975 was determined using triplicate injections of the calibration standards.

Some experiments contained neomycin at elevated concentrations (1.0, 6.0 and 15 g neomycin L^−1^). The highest external calibration concentration was 34 mg neomycin L^−1^. To be able to quantify neomycin in these experiments, dilutions of SPE-cleaned collected fractions (200 µL) with MilliQ water were made. These dilutions were made in such a manner that the maximum end concentration would be 34 mg L^−1^. The initial concentration: theoretical final volume relations were as follows; 1.0 g L^−1^: 5882 µL, 6.0 g L^−1^: 35,294 µL, 15 g L^−1^: 88,235 µL. The SPE recoveries for the elevated neomycin concentrations were proven to be close to 100% by dividing the initial concentrations in Figs. 4f, g, h and 6c, d, e with the final volumes (5882, 35,294 and 88,235 µL) and then multiplying with 200 µL. The majority of the back calculated concentrations were 33 mg neomycin L^−1^.

#### Qualitative analysis of biodegradation products

Molecular formulas of possible oxidation products of neosamine, 2-DOS, D-ribose, neobiosamine and neamine (see Fig. 1) including ketones, aldehydes and acids were added to a PCDL-database which was integrated in the MassHunter software. Also, a number of compounds, previously identified as fungal metabolites and secondary metabolites (small organic molecules that are not directly involved in primary metabolism processes) were added to the database. These were glucuronic acid, fumaric acid, gluconic acid and citric acid (metabolites) and itaconic acid and ribitol (secondary metabolites). After a finalized run, the database was searched and compounds were preliminarily identified, based on mass errors, isotopic abundances, and spacings. Ions with mass errors and scores of < 2.5 ppm and > 80% respectively, were considered. An alternative approach was to search in the TIC-chromatograms for ions that were missing from the blanks. The “Generate Formula From Peak Spectra” algorithm was used and the ions with mass errors and scores of < 2.5 ppm and > 80%, respectively, were further evaluated using the software ChemSpider [29].

#### Statistical analysis

In Table 2, the calculations of the accurate and exact masses in MS were facilitated by the use of Agilent MassHunter software (v. 06.00). Furthermore, using this software, elemental compositions were suggested for detected ions. Excel, Microsoft Office Version 2110, was used for regression analysis purposes to construct least square fitted (i) Calibration curves and (ii) Kinetic models. The Pearson product-moment correlation coefficient (*r*) was used to determine linearity. Excel was also used to calculate confidence intervals to confirm whether any biodegradation occurred or not.

**Table 2:** Summary of the accurate mass measurements of hydrolysis and oxidation products of neomycin, as determined for their abundant adduct ions using UHPLC-Q-TOF MS

Compound	Elemental	Ion species	Accurate	Exact	Mass error		*t*_R_(min)
	composition		mass (*m/z*)	mass (*m/z*)	mDa	ppm	
Neomycin	C_23_H_46_N_6_O_13_	[M+H]^+^	615.3206	615.3201	− 0.5	− 0.8	4.1
A (D-ribose)	C_5_H_10_O_5_	[M+H-H_2_O]^+^	133.0498	133.0501	0.3	2.2	4.1
B	C_5_H_8_O_5_	[M+NH_4_-H_2_O]^+^	148.0608	148.0610	0.2	1.4	3.3
C	C_5_H_6_O_5_	[M+H-H_2_O]^+^	129.0185	129.0188	0.3	2.3	3.3
D	C_5_H_8_O_6_	[M+H-H_2_O]^+^	147.0291	147.0293	0.2	1.4	3.3

The elemental compositions are shown in their uncharged state. Data for detected ions correspond to acquisitions obtained in full-scan mode. Most abundant peak *t*_R_

#### Kinetics

Free radical processes which are present in the extracellular degradation of chemicals using white rot fungi can at co-metabolic conditions follow pseudo-first order kinetics [30, 31]. See Eq. (2).

2 $$\ln C_{t} = - k^{\prime } t + \ln C_{0}$$

where *C*_*t*_ is the concentration at time *t*. *k*^´^ is the degradation rate constant and C_0_ is the initial concentration.

When the target substance is present at a high concentration level, the reaction can obey zero order kinetics [32]. See Eq. (3). The reaction rate is thus independent of the target compound concentration level. Zero-order reactions are only applicable for a narrow region of time. After this time period, other kinetic models are better applicable.

3 $${C}_{t}=-kt+ {C}_{0}$$

#### Evaluation of neomycin biodegradation curves

The neomycin biodegradation curves (excluding the four HKCs) were plotted and compared with each other. The results from the last collected fractions at 168 h, were used to determine the final removal degrees (%). Furthermore, these results were also used to calculate the total amount of neomycin that was biodegraded in each experiment. See the general Eqs. (4) and (5).

4 $$Removal \, degree\left( {{\text{R}}\% } \right)\, = \,100 - \left( {\frac{{C_{t} \times 100}}{{ C_{01} }}} \right)$$

where *C*_*t*_ is the neomycin concentration at time *t* and *C*_01_ is the initial neomycin concentration in the analyzed sample.

5 $$Removal \, amount = \frac{{R\left( \% \right) \times 0.3 \times C_{02} }}{100}$$

where 0.3 is the liquid volume (*L*) in the E-flask. *C*_02_ is the initial neomycin concentration (mg L^−1^) in the biodegradation experiments (four different concentrations).

A matrix which consisted of these two measures of the biodegradation and the carbon and nitrogen contents in the experiments were analyzed using Principal Component Analysis (PCA) to (i) determine whether the biodegradation could be classified as co-metabolism or nutrition mediated and (ii) possibly determine the most favourable conditions for the biodegradation. The raw data that were used in PCA were centered and univariately scaled [33].

## Results and discussion

### Co-metabolism

**Fig. 2:**
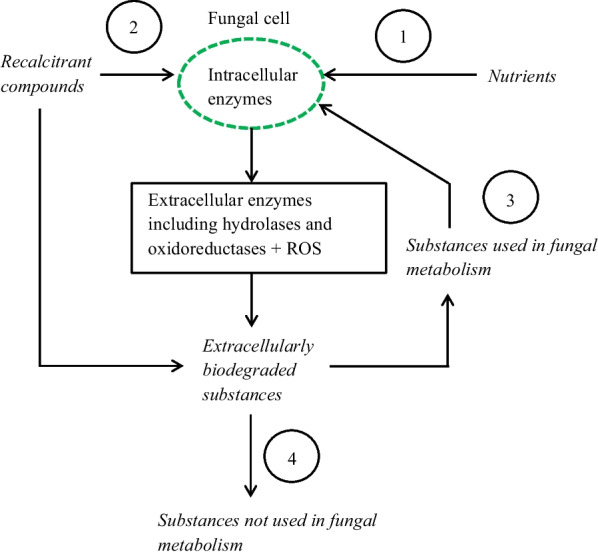
Co-metabolism in which the fungal cell is supplied with nutrients (**1**) and recalcitrant compounds which either are transported through the fungal cell walls and plasma membranes and used in the metabolism of the fungus (**2**) or in their extracellularly catalyzed biodegraded forms (**3**). Biodegraded substances not used in the fungal metabolism are denoted by (**4**)

The term co-metabolism is in the present paper defined as “the transformation of a substrate in the obligate presence of known growth substrates” It differs slightly from a previous definition of co-metabolism which excludes that the substrate is used as nutrient “the transformation of a non-growth substrate in the obligate presence of a growth substrate or another transformable compound” [34]. The suggested modified definition includes two alternative approaches; the transformed (biodegraded) compound is (i) used or (ii) not used by the fungus in its metabolism including the biosynthesis of secondary metabolites. The concept co-metabolism is included in a reaction scheme in which different fungal reaction paths are described including extracellular and intracellular enzymes (see Fig. 2). In the scheme, a hypothetical co-metabolism of recalcitrant compounds like neomycin is shown. Reactive oxygen species (ROS) are oxidants that participate in the cleavage of ether groups. ROS contain H_2_O_2_, •OH, ROO• and •OOH radicals. Their presence in fungal species belonging to the phylum Basidiomycota is well documented [35].

One reason why an extracellular enzymatically catalyzed compound is not metabolized by the fungus can be that the substance is not soluble [36]. Furthermore, it cannot be excluded that the chemical structure or size of the compound does not allow the fungus to metabolize it [37].

### HKC-results

**Fig. 3:**
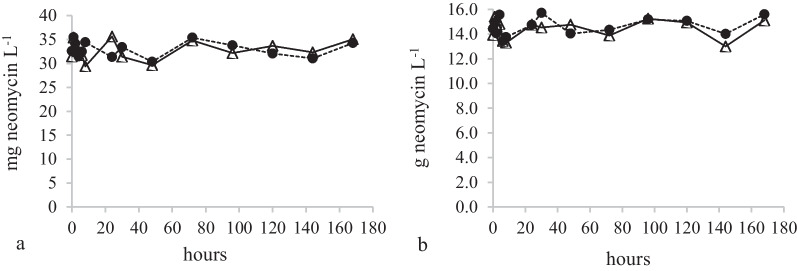
HKC-experiments NSHKCTv and NSHKCRe (**a**) and NeoHKCTv and NeoHKCRe (**b**). *T. versicolor* (•), *R ericae* (Δ)

No neomycin adsorption could be observed in the HKC results for experiments NSHKCTv and NSHKCRe which contained neomycin at a concentration level of 34 mg L^−1^ in the presence of NS. The HKC results for the highest neomycin concentration levels (see Table 1) excluding other nutrients (NeoHKCTv and NeoHKCRe) did not either indicate any neomycin adsorption (See Fig. 3).

The lack of neomycin adsorption in the HKC experiments might be explained by the positive charges of neomycin and chitin which is one of the components in the fungal cell wall. At pH 4.0, we can expect that the amino groups in chitin, which is a biopolymer of N-acetylglucosamine are mainly protonated [38]. This will lead to a repulsive electrostatic force between neomycin and chitin. Furthermore, at this pH, a protonation of glycoproteins which also are present in the fungal cell walls can occur.

### *T.versicolor* neomycin biodegradation

**Fig. 4:**
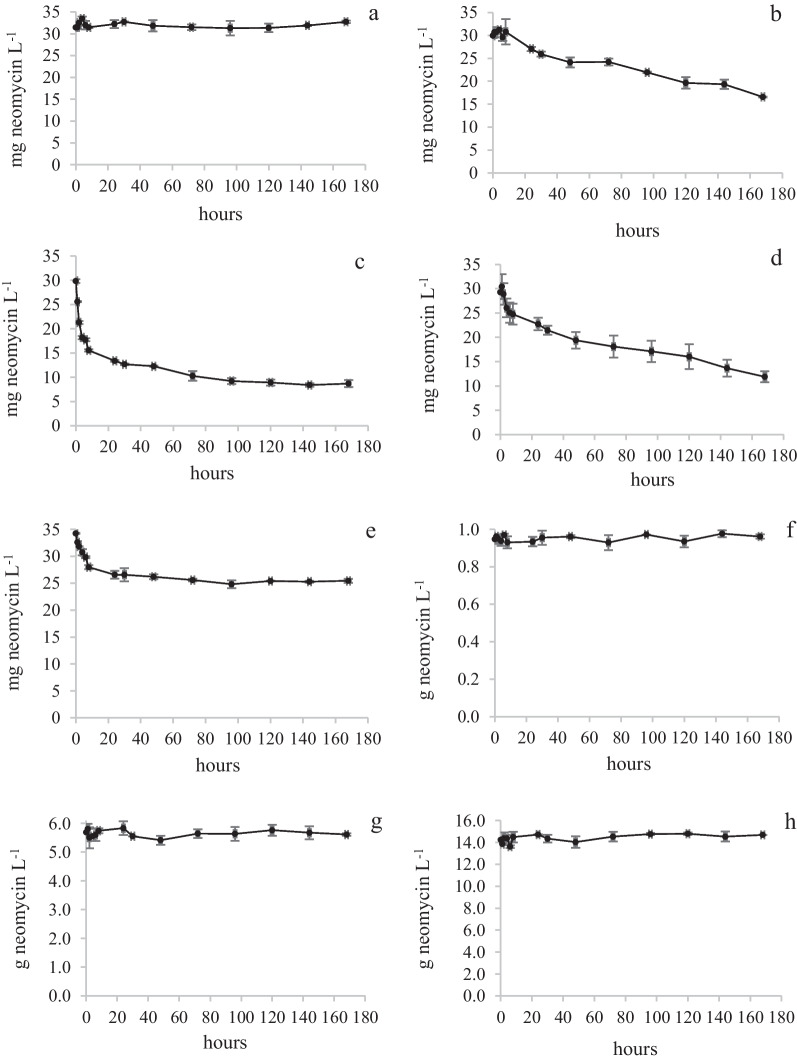
Biodegradation curves of *T.versicolor*. NS1Tv (**a**), NS2Tv (**b**), NS3Tv (**c**), NS4Tv (**d**), Neo1Tv (**e**), Neo2Tv (**f**), Neo3Tv (**g**) and Neo4Tv (**h**). Mean values and absolute spread

Measurements of the fungal biomass (BM) during a biodegradation study is of interest since it can give information of to what extent, the nutrients and other chemicals are used by the fungus for its growth. A low C:BM ratio indicates a high utilization degree. In the present study, it was realized that it is difficult to measure BM of immobilized fungal mycelia (*T. versicolor*). Therefore, it was decided to use the concept “initial biomass”. The biodegradation curves are shown in Fig. 4.

**Fig. 5:**
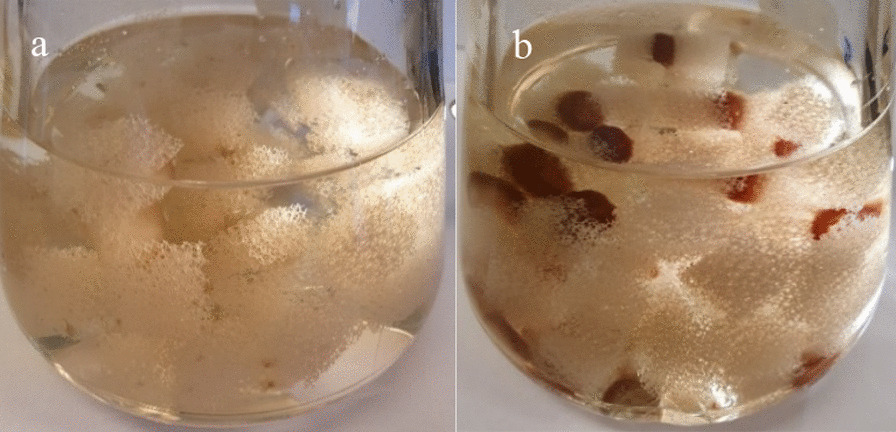
Immobilized mycelia of *T. versicolor* in NS4Tv (**a**) and Neo1Tv (**b**) experiments

The results show that the highest removal degree (approximately 74%) was achieved in experiment NS3Tv (Fig. 4c and Additional file 2: Appendix 2). In this experiment, the glucose and ammonium tartrate concentrations in NS were 1.20 and 0.90 g L^−1^, respectively (see Table 1). At the lowest NS-concentrations (NS1Tv), the neomycin removal degree was only approximately 4%. The C_tot_:BM ratio for NS1Tv was 1.0 while the corresponding ratio for NS3Tv was 18.6 (see Additional file 1: Appendix 1). The removal degree was thus affected by the concentration of NS. Excluding NS at a neomycin concentration of 34 mg L^−1^ (Neo1Tv), the removal degree was approximately 25%, and this concentration level was achieved already after 50 h. It was observed that the colour of the immobilized mycelia that was still in contact with agar plug residues originating from the inoculation step, turned red after two days. This is shown in two photographs that were taken on uncoloured *T. versicolor* mycelia in NS4Tv (Fig. 5a) and Neo1Tv (Fig. 5b). The colour change indicates an incapability of *T. versicolor* to continuously use neomycin as nutrient. This was confirmed in the Neo2Tv, Neo3Tv and Neo4Tv experiments. In those experiments, the colour change was also present. Generally, a colour change of mycelia does not necessarily indicate that the growth is inhibited. The reason can be that other biodegradation mechanisms occur [39]. However, in the present study, it was shown that the colour change only occurred in those experiments in which NS was excluded.

The reason for the neomycin decline in Neo1Tv (which actually was considerably higher than in NS1) during the first 50 h can be that *T.versicolor* in Neo1Tv initially uses neomycin as nutrient or alternatively bioaccumulates it. Then, at approximately 50 h, the metabolism of the fungus is negatively affected by the aminoglycoside.

In all performed experiments, some amounts of NS are most certainly left in the PUF pores after the decanting procedure (see section “Experimental design”). The 54 PUF pieces (each weighing approximately 50 mg) contains a volume of 53 mL (max 98% porosity). However, the reason why the neomycin removal was higher in Neo1Tv compared with NS1Tv cannot be explained by NS residues from the inoculation period since they were present in both experiments.

#### Kinetics

Previously, the kinetics of the biodegradation of neomycin using *T.versicolor* has been investigated under co-metabolic conditions [14], in which the initial neomycin concentration was approximately 1/3 of the concentration in the present study. In the previous study, the biodegradation obeyed zero order kinetics at start followed by pseudo-first order kinetics after a concentration decline. The biodegradation of neomycin in Neo1Tv obeyed pseudo-first order kinetics during the first 8 h with correlation coefficients (Pearson product movement, *r*) of 0.9791 and 0.9826, respectively for the duplicate biodegradation experiments. A Pearson correlation coefficient > 0.9 is defined as a strong correlation and indicate that the reaction order is relevant [40]. The biodegradation of neomycin in NS2Tv and NS4Tv were also explained by pseudo-first order kinetics with *r*-values ranging from 0.9593 to 0.9759. However, in NS3Tv, only the biodegradation results from the first 8 h could be similarly explained with less accurate *r*-values of 0.9467 and 0.9473. See Additional file 3: Appendix 3. Pseudo-first order kinetics can be explained by Michaelis Menten kinetics in which the substrate (neomycin) concentration [*S*] < *K*_*m*_. In our case, *K*_*m*_ is not known. The mechanism behind the biodegradation is not investigated although degradation products previously [14] and in the present study are tentatively identified.

In the Neo2-4Tv experiments, no neomycin decline was visible by eye (see Fig. 4 f,g,h). The confidence intervals of the mean (*p* < 0.05) for each single curve covering the time courses were calculated and compared with the end neomycin concentrations at 168 h. Diluted concentrations were used in the calculations (see section “Quantitative analysis of neomycin”). For Neo2Tv, the confidence intervals and end concentrations for the duplicate experiments were; 32 ± 0.5, 32 and 32 ± 0.7, 33 respectively. For Neo3Tv, the corresponding values were; 32 ± 0.8, 32 and 32 ± 0.7, 32 respectively. Finally, the values for Neo4Tv were 33 ± 0.6, 33 and 33 ± 0.7, 33 respectively. Only one of the end concentrations (Neo2Tv) was slightly outside the confidence interval of the mean. Thus, no biodegradation could be verified for Neo2-4Tv.

Conclusion: The highest removal degree and removal amount was achieved in the NS3 experiment in which the glucose and ammonium tartrate concentrations were 1.2 and 0.9 g L^−1^ respectively. The C_tot_:BM ratio was 18.6. At lower C_tot_:BM ratios (experiments NS1Tv and NS2Tv), the biodegradation decreased (see Additional file 1: Appendix 1). In NS1Tv, in particular, with a C_tot_:BM ratio of 1.0, the nutrient concentration level was not sufficient for the fungus to keep up the neomycin reduction. The reason why the removal degree did not increase in NS4Tv with a higher nutrient concentration than in NS3Tv may be the slightly elevated C_tot_:N_tot_ ratio in NS4Tv (6.4) compared with NS3Tv where the corresponding ratio was 5.1 (see Additional file 1: Appendix 1). It is previously reported that the metabolism of *T. versicolor* is positively influenced by an increased nitrogen addition (lower nutrient C:N ratio) [18].

The Neo 1 experiment results also indicated that the fungus was not able to use neomycin as nutrient during the whole experimental time course. Initially in this experiment (excluding NS) with a C_tot_:BM ratio of approximately 0.1 (see Additional file 1: Appendix 1), a neomycin removal occurred. After approximately 50 h, a colour change of mycelia was observed and after that, no further neomycin removal occurred. These results show that neomycin acts as a fungicide when NS is excluded. It was confirmed by the results from the Neo2Tv, Neo3Tv and Neo4Tv experiments in which no neomycin biodegradation could be observed, which leads to the conclusion that the higher concentration levels in these experiments are toxic to the fungus.

### *R. ericae* neomycin biodegradation

**Fig. 6:**
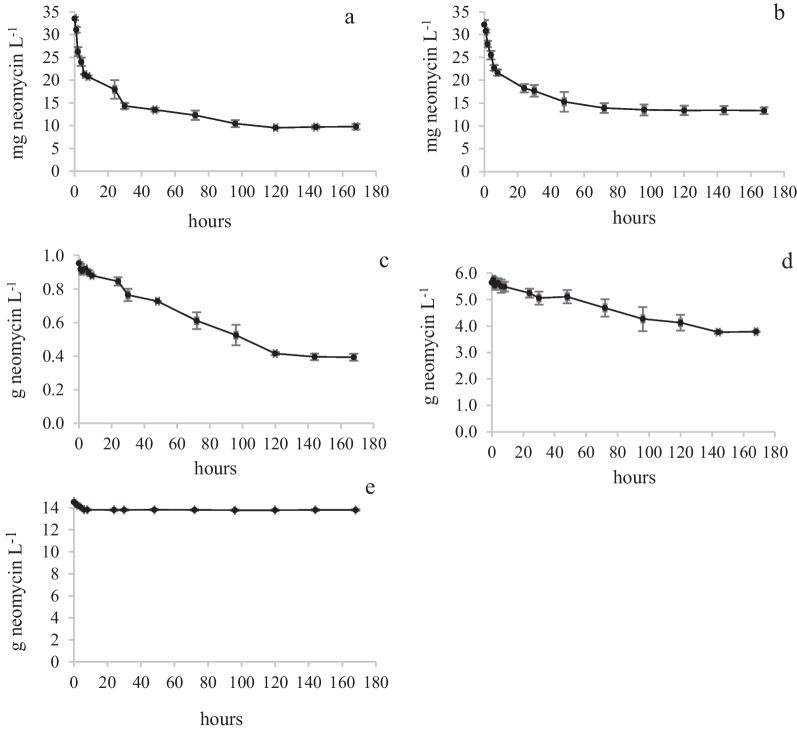
Biodegradation curves of *R.ericae*. NS4Re (**a**), Neo1Re (**b**), Neo2Re (**c**), Neo3Re (**d**), Neo4Re (**e**). Mean values and absolute spread

As for *T. versicolor*, the initial biomass concept was used in the calculation of C_tot_:BM and C_neo_:BM ratios. The biodegradation curves are shown in Fig. 6.

The results show that the highest removal degree (approximately 71%) was achieved in experiment NS4Re (Fig. 6a and Additional file 2: Appendix 2). In this experiment, NS was present with glucose and ammonium tartrate concentrations of 6.0 and 3.3 g L^−1^ respectively and a neomycin concentration of 34 mg L^−1^ (C_tot_:BM and C_neo_:BM of 10.7 and 0.06 respectively). See Additional file 1: Appendix 1. Interestingly, it was shown that it was possible for *R. ericae* to biodegrade neomycin also in the absence of NS at four different neomycin concentrations. In Neo1Re, the same C_neo_:BM ratio as in NS4Re was present. Despite the absence of NS, this particular experiment showed that the limited amount of neomycin was sufficient for the survival of the fungus. The neomycin decline in Fig. 6b (Neo1Re), can at least partly be explained by co-metabolism using residual NS from the inoculation procedure in the PUF pores. All of the *R.ericae* experiments showed that this species can use neomycin as nutrient. The removal degree was higher in the experiments in which neomycin was present at 34 mg and 1.0 g L^−1^ (NS4Re, Neo1Re and Neo2Re). See Additional file 2: Appendix 2. At the two higher neomycin concentrations, Neo3Re and Neo4Re (6.0 and 15 g L^−1^), it was shown that considerable amounts of neomycin were removed although the removal degrees were declining. It can be explained by a limited ability of the fungus to metabolize these high concentrations of neomycin. Even if the NeoHKCRe experiment in which 15 g neomycin was added to heat treated fungi, did not indicate any neomycin adsorption to the dead biomass, it cannot be excluded that neomycin to some extent can be adsorbed to living fungal cells. In a study performed on the fungal species *Funalia trogii,* in which the adsorption of phenols was investigated, it was concluded that heat treatment not only removed functional groups on the fungal cell walls, but also decreased their surface area [41].

### Kinetics

The biodegradation curves for the NS4Re and Neo1Re experiments showed a sharp initial decline (see Fig. 6 a and b). The declines in the two duplicates of NS4Re were not well explained, neither by zero or first order kinetics. However, by applying zero order kinetics on the first 8 h period, correlation coefficients of 0.9120 and 0.9637 were obtained. Similarly for the Neo1Re experiments in which solely neomycin was present, it was only possible to explain the first 8 h time course. For these experiments, pseudo-first order kinetics was the better choice with *r*-values of 0.9793 and 0.9885. As mentioned in a previous study [14], in which the biodegradation of neomycin using *T.versicolor* was investigated, biodegradation can be facilitated by extracellular, intracellular or reactive oxygen species which renders the interpretation of declining concentrations difficult. The biodegradation curves for Neo2Re obeyed pseudo-first order kinetics during the whole time course with *r*-values of 0.9742 and 0.9863. For Neo3Re, with an initial neomycin concentration of 6 g L^−1^, zero order kinetics was the better choice with *r*-values of 0.9574 and 0.9855. See Additional file 3: Appendix 3.

Conclusion: The highest removal degree was achieved in the NS4Re experiment which included NS. The C_tot_:BM ratio was 10.7. In the experiment Neo1Re which did not contain NS, the removal degree was high, even if the C_tot_:BM ratio (C_neo_:BM ratio) was considerably lower (0.06). The conclusion is that the fungus can survive and remove neomycin in the absence of NS. In the Neo2Re, Neo3Re and Neo4Re experiments, with increasing C_neo_:BM ratios (see Additional file 1: Appendix 1), the removal degrees declined, thereby showing that neomycin was less efficiently metabolized at these elevated concentrations of neomycin.

### PCA evaluation of biodegradation curves

**Fig. 7:**
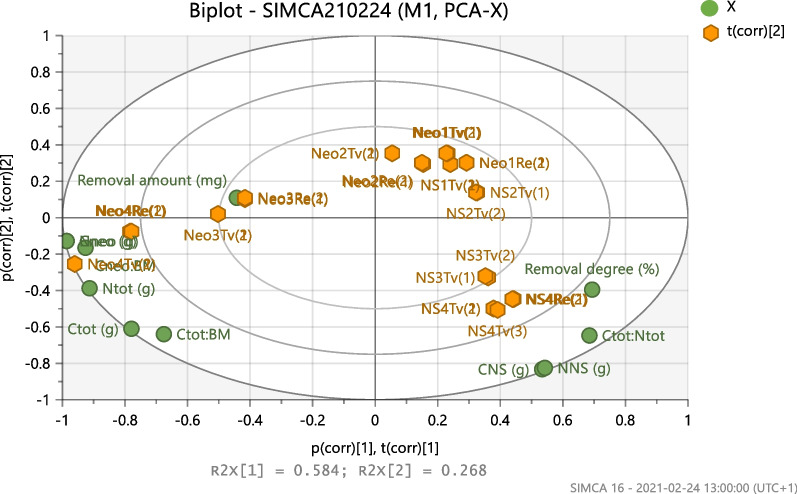
PCA-biplot including all experiments (orange) and variables (green)

In Fig. 7, a PCA-biplot is shown which includes all performed experiments (orange) including *T. versicolor* and *R. ericae*. The model matrix X variables (see section “Experimental design”) are shown in green. In Additional file 1: Appendix 1, the complete experimental matrix is shown. The biplot shows the PC scores (orange) of experiments (data) and loadings (green) of variables (vectors). The loadings in Fig. 7 include both the *T.versicolor* and the *R.ericae* experiments. This type of plot facilitates the understanding how the variables influence the experiments. In Fig. 7, principal components (PC) 1 and 2 were used. The cumulative explanation (R2) and predictability (Q2) of the model was 0.85 and 0.78 respectively.

Some general conclusions can be drawn from the plot.

- A high neomycin removal degree is negatively correlated to the concentrations of neomycin (*T. versicolor* and *R. ericae*).
- The neomycin removal degree is dependent on C_NS_ and N_NS_ (*T.versicolor*)
- The removal degree is negatively correlated to the removal amount (*R.ericae*)

Separate PCA-models for *T.versicolor* and the *R. ericae* were also performed. See Additional file 4: Appendix 4. In the *T. versicolor* model, the variables “removal degree” and “removal amount” perfectly coincided. The neomycin degradation was dependent on the NS concentration at 34 mg neomycin L^−1^ using co-metabolic conditions. In the *R. ericae* model, these variables were opposed to each other. *R.ericae* could biodegrade large initial amounts of neomycin (≥ 1.0 g L^−1^), but the fungus was not able to metabolize the supplied neomycin as efficiently as when the compound was added at lower concentrations.

### C_neo_:BM ratio for *R. ericae*

In the section “PCA evaluation of biodegradation curves”, the initial BM values for *T. versicolor* and *R. ericae* were used in the PCA-model. They were calculated from the weights of the mycelia that were cultivated during a time period of two weeks and then used for inoculation purposes. Since *R. ericae* was not immobilized on PUF, it was feasible to measure the growth of its biomass using the previously described experimental conditions (see Table 1). In addition, this investigation also included weight measurements on BM in experiments in which no neomycin but solely NS was present. In Additional file 5: Appendix 5, the raw data is presented including the growth of *R.ericae* at different neomycin concentrations. It was shown that in those experiments in which no NS was present, the lowest C_neo_:BM ratio (approximately 0.03) was present at a neomycin concentration of 34 mg L^−1^. At higher neomycin concentrations, the C_neo_:BM ratio increased. At a concentration of 15 g L^−1^, the ratio was > 5. The conclusion is that neomycin is most efficiently metabolized at a concentration of 34 mg L^−1^. These experiments proved that this fungal species can use both NS and neomycin as nutrients (see Additional file 5: Appendix 5).

### Biodegradation mechanisms

**Fig. 8:**
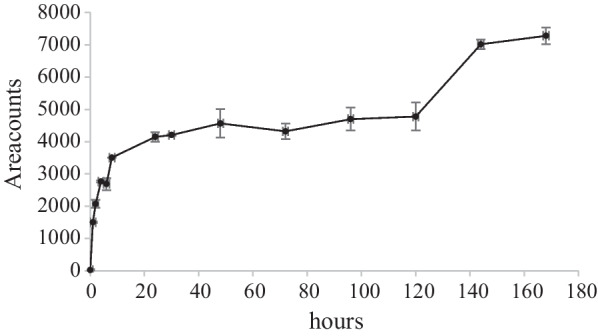
Time course of neosamine oxidation product at *m/z* 157.0163 in NS4Tv (*n* = 2). Mean values and absolute spread

In a recently published paper [14], in which *T. versicolor* was the solely investigated fungal species, it was discovered that under co-metabolic biodegradation conditions, and at an initial neomycin concentration of 10 mg L^−1^, the tentatively identified biodegradation products were oxidation products of neosamine (see Fig. 1). In the present investigation, in which not only higher initial neomycin concentrations (34 mg L^−1^) but also non co-metabolic conditions were applied (absence of NS), the biodegradation patterns were investigated. In the experiments that included *T. versicolor*, only one biodegradation product of neomycin was found in NS4Tv (highest concentration of NS) (see Table 1). This compound with an elemental neutral composition of C_6_H_10_N_2_O_4_ was tentatively identified by its [M + H-H_2_O]^+^ ion at *m/z* 157.0613. The exact mass for this ion is 157.0610. The calculated mass error was -1.9 ppm. The retention time (*t*_R_) was 3.1 min which can be compared with neomycin at *t*_R_ 4.1 min. The biodegradation product has previously been determined as an oxidation product of neosamine [14]. The shorter retention time (compared with neomycin) is most probably due to the two keto-groups. In Fig. 8, the time-course of this compound in the NS4Tv experiment is shown. The steep increase of the oxidation product during the first 8 h can be correlated with the fast neomycin decrease during the same time period (see Fig. 4). The neomycin decline during the whole time course was best explained by pseudo-first order kinetics. However, the response variations of the oxidation product in Fig. 8, could not be explained by neither zero, nor pseudo-first order kinetics. The reason for this may be its role as an intermediate in the biodegradation of neomycin.

The extracted ion chromatogram (EIC) showed the presence of two peaks which can be explained by the six isomeric forms of this compound (neosamine B and C and three possible keto-group positions in each neosamine form). These two peaks were co-integrated.

The reason why no oxidation products were found in the other experiments which included *T. versicolor* may be that the particular experimental conditions in those experiments in which the NS-concentrations were lower, did not favour the formation of these compounds, but other substances that not were easily detected. It is known that biodegradation experiments that are performed under co-metabolic conditions are affected by variations in substrate:NS ratios [42].

**Fig. 9:**
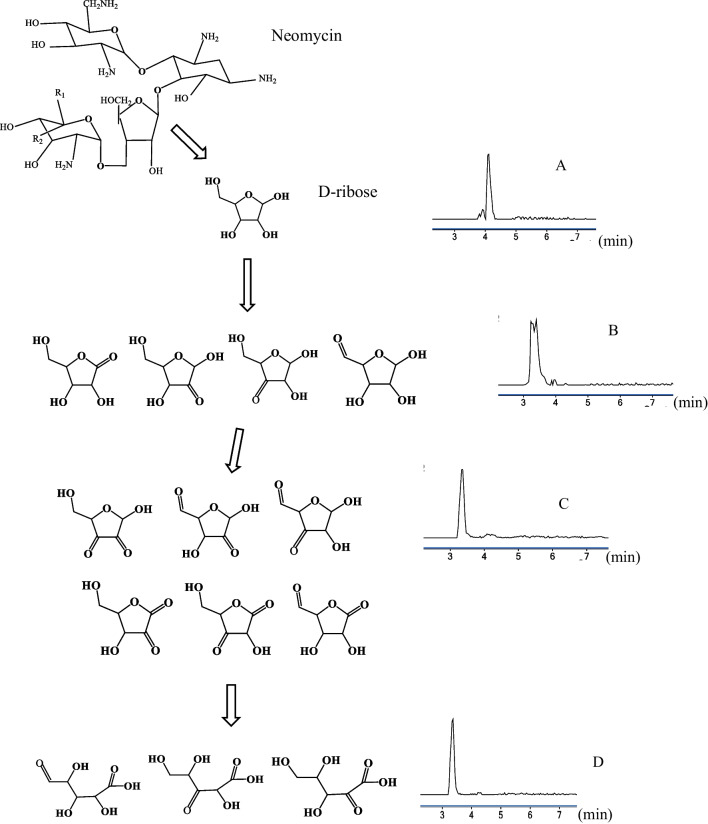
Proposed biodegradation pattern of neomycin using *R. ericae*, where (**B**)–(**D**) show possible structural isomers of oxidation products of D-ribose (**A**). The tentative identities of the oxidation products were verified by UHPLC-Q-TOF MS analyses. Extracted ion chromatograms (Neo1Re, 168 h) at *m/z* 133.0498 (**A**), 148.0608 (**B**), 129.0185 (**C**) and 147.0291 (**D**)

Interestingly, in the *R.ericae* experiments, no neosamine derived biodegradation products were found. Neither were oxidation products of 2-DOS observed. However, oxidation products of D-ribose were tentatively identified in all experiments. See Fig. 9. The oxidation products are denoted (B-D). The elemental compositions are expressed in their neutral forms. Both aldehydes and ketones can be present, but aldehydes are typically less stable. The initial hydrolysis of neomycin in NS4Re may be originated by the oxidoreductase glucose oxidase which is known to be secreted by *R.ericae* under co-metabolic conditions using glucose as carbon source. Glucose oxidase oxidizes carbohydrates to lactones under the production of H_2_O_2_ which can lead to the formation of hydroxyl radicals that can hydrolyze ether bonds [43]. However, since Neo1Re, Neo2Re, Neo3Re and Neo4Re were performed in the absence of glucose (non-metabolic conditions), the hypothesized pathway is not plausible for those experiments if we exclude the possible formation of glucose via gluconeogenesis.

D-ribose (A) with the composition C_5_H_10_O_5_ was tentatively identified by the ion species [M + H-H_2_O]^+^ at *m/z* 133.0498 The exact mass is 133.0501 and the mass error is 2.2 ppm. A solution of D-ribose dissolved in MilliQ water at a concentration of 10 mg L^−1^ was injected, using the default instrumental conditions including an injection volume of 15 µL. The substance was identified at a *t*_R_ of 4.1 min at *m/z* 133.0500, thus proving the presence of D-ribose in NS4Tv. Oxidation products (B), with the composition C_5_H_8_O_5_ were identified by the ion species [M + NH_4_-H_2_O]^+^ at *m/z* 148.0608. The exact mass is 148.0610 and the mass error is 1.4 ppm. Oxidation products (C) with the composition C_5_H_6_O_5_ were identified by the ion species [M + H-H_2_O]^+^ at *m/z* 129.0185. The exact mass is 129.0188 and the mass error is 2.3 ppm. Oxidation products (D) with the composition C_5_H_8_O_6_ were identified by the ion species [M + H-H_2_O]^+^at *m/z* 147.0291. The exact mass and mass error is 147.0293 and 1.4 ppm respectively. In Table 2, a summary of the accurate mass measurements and retention times on hydrolysis products and oxidation products that were detected in full scan mode is shown.

The retention time of D-ribose coincides perfectly with the retention time of neomycin (4.1 min.). By performing analyses of neomycin calibration standards, it was confirmed that *m/z* 133.0498 was not an ion-source fragment ion or impurity in neomycin, since this ion was only present in the experiments in which *R. ericae* was included.

**Fig. 10:**
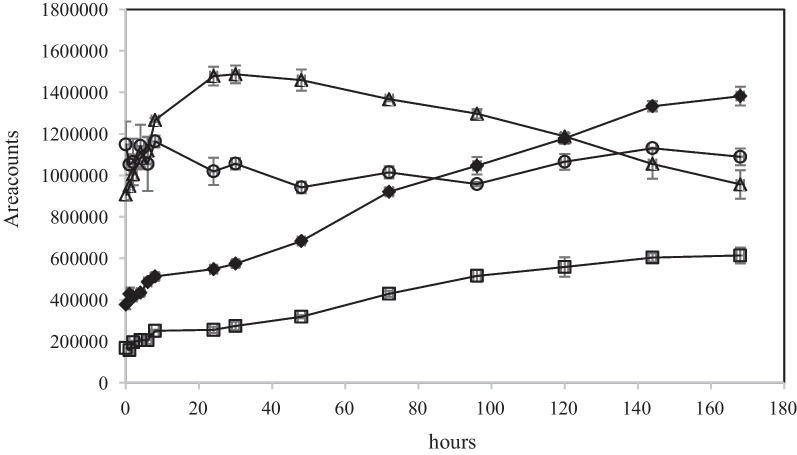
Time course of oxidation products A – D (see Fig. 9) at *m/z* 133.0498 (**A**), 148.0608 (**B**), 129.0185 (**C**) and 147.0291 (**D**). Symbols: ○ (A), Δ (B), ♦ (C), □ (D) in NS4Re experiment. Mean values and absolute spread (*n* = 2)

**Fig. 11:**
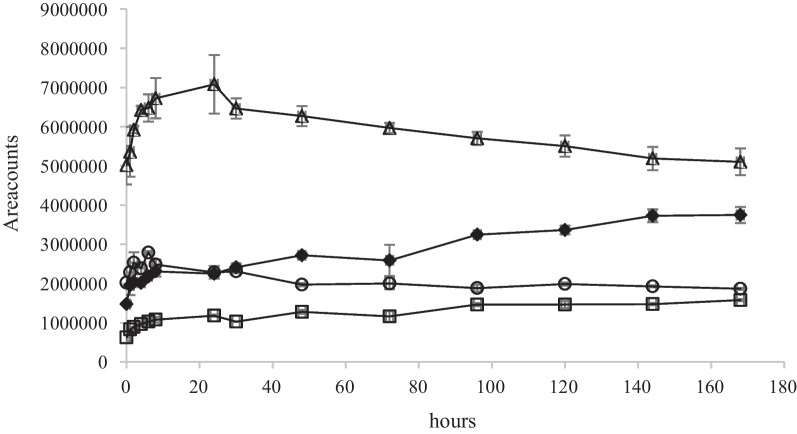
Time course of oxidation products A – D (see Fig. 9) at *m/z* 133.0498 (**A**), 148.0608 (**B**), 129.0185 (**C**) and 147.0291 (**D**). Symbols: ○ (A), Δ (B), ♦ (C), □ (D) in Neo1Re experiment. Mean values and absolute spread (*n* = 2)

The time course of biodegradation products A – D in NS4Re and Neo1Re are shown in Fig. 10 and in Fig. 11 respectively. The responses of D-ribose (A) were more or less stable in the two experiments. The intensity levels at the start of the experiments are explained by the immediate start of the biodegradation processes. It must also be mentioned that the first fractionating occurred approximately 0.2 h after the addition of neomycin.

The time courses in these two experiments, are similar with respect to the decline of oxidation products B and increase of oxidation products C. However, the intensities of the ions in the Neo1Re experiment are approximately 5 times higher than in the NS4Re experiment in which NS was present. It can be explained by the co-metabolic (NS4Re) and non co-metabolic (Neo1Re) conditions under which the experiments were conducted. This increase is not correlated with the removal degree (See Fig. 6 and Additional file 2: Appendix 2). In the three *R. ericae* experiments in which the initial neomycin concentration were higher (1.0, 6.0 and 15 g L^−1^), the same biodegradation products which are shown in Fig. 9 could be tentatively identified. The MS responses were approximately at the same levels as in the NS4Re experiments. However, no time course related concentration changes could be seen. It may be so that this difference regarding formation of oxidation products between the *R.ericae* experiments can be correlated with the rapidness of the neomycin decline in the performed experiments. In NS4Re and Neo1Re, the biodegradation curves showed a more steep decline. See Fig. 6. The reason for this decline variation can be found in the different conditions by which the experiments were performed (see Additional file 1: Appendix 1).

## Conclusions

It was investigated under which nutritional conditions the fungal species *T.versicolor* and *R. ericae* can biodegrade the aminoglycoside neomycin. The biodegradation experiments showed that *T. versicolor* needs external nutrients. It cannot survive on sole neomycin. Furthermore, the neomycin removal degree is dependent on the concentration of the external nutrient solution (NS). The *T.versicolor* mediated biodegradation can at high NS concentrations be traced to the neomycin hydrolysis product neosamine. Contradictory to *T. versicolor*, it was shown that *R.ericae* can survive on sole neomycin. At selected conditions, it was found that the neomycin biodegradation pattern using *R.ericae* can be traced to the oxidation of the neomycin hydrolysis product D-ribose. The results in this study are of importance in future biodegradation experiments with emphasis not only on recombinant protein production using *E.coli*, but also on other applications where a removal of more concentrated recalcitrant compounds is desirable.

## Supplementary Information

**Additional file 1.** Appendix 1.

**Additional file 2.** Appendix 2.

**Additional file 3.** Appendix 3.

**Additional file 4.** Appendix 4.

**Additional file 5.** Appendix 5.

## Data Availability

The datasets use and/or analysed during the current study are available from the corresponding author on reasonable request.

## References

[1] Löffler D, Ternes TA (2003). Analytical method for the determination of the aminoglycoside gentamicin in hospital wastewater via liquid chromatography- electrospray-andem mass spectrometry. J Chromatogr A.

[2] Zawilla NH, Diana J, Hoogmartens J, Adams E (2006). Analysis of neomycin using an improved liquid chromatographic method combined with pulsed electrochemical detection. J Chromatogr B.

[3] Waksman SA, Lechevalier HA, Harris DA (1949). Neomycin-production and antibiotic properties. J Clin Investig.

[4] Prokhorova I, Altman RB, Djumagulov M, Shrestha JP, Urzhumtsev A, Ferguson A, Chang CWT, Yusupov M, Blanchard SC, Yusupova G (2017). Aminoglycoside interactions and impacts on the eukaryotic ribosome. Proc Nat Acad Sci.

[5] Chang CWT, Takemoto JY (2014). Antifungal amphiphilic aminoglycosides. Medchemcomm.

[6] Junker T, Radka A, Knackerv T, Kümmerer K (2006). Biodegradability of ^14^C- labelled antibiotics in a modified laboratory scale sewage treatment plant at environmentally relevant concentrations. Env Sci and Technol.

[7] Mitani K, Kataoka H (2006). Determination of fluoroquinolones in environmental waters by in-tube solid-phase microextraction coupled with liquid chromatography-tandem mass spectrometry. Anal Chim Acta.

[8] Tahrani L, van Locco J, Mansour HB, Reyns T (2016). Occurence of antibiotics in pharmaceutical industrial wastewater, wastewater treatment plant and sea waters in Tunisia. J Water Health.

[9] Jury KL, Vancov T, Stuetz RM, Khan SJ, Mendez- Vilas A (2010). Antibiotic resistance dissemination and sewage treatment plants. Current research, technology and education topics in applied microbiology and microbial biotechnology.

[10] Wesgate R, Evangelista C, Atkinson R, Shepard A, Adegoke O, Maillard JY (2020). Understanding the risk of emerging bacterial resistance to over the counter antibiotics in topical sore throat medicines. J Appl Microbiol.

[11] Kappell AD, DeNies MS, Ahuja NH, Ledeboer NA, Newton RJ, Hristova KR (2015). Detection of multi drug resistant *Escherichia coli* in the urban waterways of Milwaukee. WI Front Microbiol.

[12] Hauschild T, Sacha P, Wieczorek P, Zalewska M, Kaczyńska K, Tryniszewska E (2008). Aminoglycosides resistance in clinical isolates of Staphylococcus aureus from a university hospital in Bialystok Poland. Folia Histochem Cytobiol.

[13] Gbylik-Sikorska M, Posyniak A, Sniegocki T, Zmudzki J (2015). Liquid Chromatography-tandem mass spectrometry multiclass method for the determination of antibiotics residues in water samples from water supply systems in food-producing animal farms. Chemosphere.

[14] Stenholm Å, Hedeland M, Pettersson CE (2022). Neomycin removal using the white rot fungus *Trametes versicolor*. J Env Sci Health Part A.

[15] Ferreira JA, Varjani S, Taherzadeh J (2020). A critical review on the ubiquitous role of filamentous fungi in pollution mitigation. Curr Pollut Rep.

[16] Gao D, Zeng Y, Wen X, Qian Y (2008). Competition strategies for the incubation of white rot fungi under non-sterile conditions. Proc Biochem.

[17] Snyder L, Champness W. Macromolecular Synthesis in *Gene Expression in Molecular genetics of bacteria*, 2nd ed. chapter 2, Washington D.C, USA: ASM Press; 2003

[18] Rousk J, Bååth E (2007). Fungal and bacterial growth in soil with plant materials of different C/N ratios. FEMS Microbiol Ecol.

[19] Pedroza-Rodríguez M, Rodríguez-Vázquez R (2013). Optimization of C/N ratio and inducers for wastewater paper industry treatment using Trametes versicolor immobilized in bubble column reactor. J Mycol.

[20] Stenholm Å, Backlund A, Holmström S, Backlund M, Hedeland M, Fransson P (2021). Survival and growth of saprotrophic and mycorrhizal fungi in recalcitrant amine, amide and ammonium containing media. PLoS ONE.

[21] Olicón-Hernández DR, González-López J, Aranda A (2017). Overview of the biochemical potential of filamentous fungi to degrade pharmaceutical compounds. Front Microbiol.

[22] Leake JR, Read DJ (1990). Proteinase activity in mycorrhizal fungi, II. The effects of mineral and organic nitrogen sources on induction of extracellular proteinase in Hymenoscyphus ericae (Read) Korf & Kernan. New Phytol.

[23] Bending GD, Read DJ (1997). Nitrogen mobilization from protein polyphenol complex by ericoid and ectomycorrhizal fungi. Soil Biol Biochem.

[24] Martino E, Morin E, Grelet G, Kuo A, Kohler A, Daghino S, Barry KW, Cichocki N, Clum A, Dockter RB, Hainaut M, Kuo RC, LaButti K, Lindahl BD, Lindquist EA, Lipzen A, Khouja HR, Magnuson J, Murat C, Ohm RA, Singer SW, Spatafora JW, Wang M, Veneault-Fourrey C, Henrissat B, Grigoriev IV, Martin FM, Perotto S (2018). Comparative genomics and transcriptomics depict ericoid mycorrhizal fungi as versatile saprotrophs and plant mutualists. New Phytol.

[25] Libra JA, Borchert M, Banit S (2003). Competition strategies for the decolorization of a textile-reactive dye with the white-rot fungi *Trametes versicolor* under non-sterile conditions. Biotech Bioeng.

[26] Lu Y, Yan L, Wang Y (2009). Biodegradation of phenolic compounds from coking wastewater by immobilized white rot fungus Phanerochaete chrysosporium. J Hazard Mat.

[27] Ullah MA, Kadhim H, Rastall RA, Evans CS (2000). Evaluation of solid substrates for enzyme production by Coriolus versicolor, for use in bioremediation of chlorophenols in aqueous effluents. Appl Microbiol Biotech.

[28] Stenholm Å, Hedeland M, Arvidsson T, Pettersson CE (2018). Identification of leachables from *Trametes versicolor* in biodegradation experiments. Trends Green Chem.

[29] ChemSpider. Chemical structure database, Royal Society of Chemistry; 2020 http://www.chemspider.com Accessed 18 May 2020.

[30] Barr DP, Aust SD (1994). Pollutant degradation by white rot fungi. Rev Environ Contamin Toxicol.

[31] Dhiman N, Chaudhary S, Singh A, Chauhan A, Kumar R (2022). Sustainable degradation of pharmaceutical waste using different fungal strains: enzyme induction, kinetics and isotherm studies. Environ Technol Innov.

[32] Huang C, Ren D, Kang C, Deng Z, Guo H, Zhang S, Zhang X (2019). Treatment of nitrogen heteocyclic compounds (NHCs) in coking wastewater by white-rot fungi. Tecnología Cienc Agua.

[33] SIMCA® software. version 16.0.0.7738. Sartorius Stedim Biotech Gmbh, Germany; 2021.

[34] Dalton H, Stirling DI (1982). Co-metabolism. Philosophical transactions of the royal society of London. Ser B Biol Sci.

[35] Hammel KE, Kapich AN, Jensen KA, Ryan ZC (2002). Reactive oxygen species as agents of wood decay by fungi. Enzyme Microbiol Technol.

[36] Gianfreda L, Rao MA (2004). Potential of extra cellular enzymes in remediation of polluted soils: a review. Enzyme Microbial Technol.

[37] Casadevall A, Nosanchuk JD, Williamson P, Rodrigues ML (2009). Vesicular transport across the fungal cell wall. Trends Microbiol.

[38] Rahman MM, Zakaria AM, Dey SC, Ashaduzzaman M, Shamsuddin SM (2017). pH controlled reversible interaction of Remazol orange with chitin. Int Lett Chem Phys Astron.

[39] Marco-Urrea E, Pérez-Trujillo M, Cruz-Morató C (2010). Degradation of the drug sodium diclofenac by Trametes versicolor pellets and identification of some intermediates by NMR. J Hazard Mat.

[40] Akoglu H (2018). User´s guide to correlation coefficients. Turk J Emerg Med.

[41] Bayramoglu G, Gursel I, Tunali Y, Arica MY (2009). Biosorption of phenol and 2-chlorophenol by *Funalia trogii* pellets. Bioresource Technol.

[42] Abdullah N, Khan AD, Ejaz N (2004). Influence of nutrients carbon and nitrogen supplementation on biodegradation of wheat straw by *Trametes versi*color. Micol Appl Int.

[43] Burke RM, Cairney JWG (1998). Carbohydrate oxidases in ericoid and ectomycorrhizal fungi: a possible source of Fenton radicals during the degradation of lignocellulose. New Phytol.

