# Supplementary material for: Investigation of neomycin biodegradation conditions using ericoid mycorrhizal and white rot fungal species
Source: BMC Biotechnol. 2022 Oct 11;22:29. doi: 10.1186/s12896-022-00759-1 (PMC9554996; doi:10.1186/s12896-022-00759-1)
Supplement: Supplementary file 2 — Additional file 2. Appendix 2. [file 12896_2022_759_MOESM2_ESM.docx]

**Appendix 2**

**Table S2** *T. versicolor*, neomycin removal characteristics after 168 hours. Mean values and absolute spread

**Table S3** *R. ericae*, neomycin removal characteristics after 168 hours. Mean values and absolute spread
