# Supplementary material for: Investigation of neomycin biodegradation conditions using ericoid mycorrhizal and white rot fungal species
Source: BMC Biotechnol. 2022 Oct 11;22:29. doi: 10.1186/s12896-022-00759-1 (PMC9554996; doi:10.1186/s12896-022-00759-1)
Supplement: Supplementary file 4 — Additional file 4. Appendix 4. [file 12896_2022_759_MOESM4_ESM.docx]

**Appendix 4**

**Figure S1** Separate PCA for *Trametes versicolor* (Class 1)

| **Component** | **R2X** | **R2X(cum)** | **Eigenvalue** | **Q2** | **Limit** | **Q2(cum)** | **Significance** | **Iterations** |
| --- | --- | --- | --- | --- | --- | --- | --- | --- |
| 0 | Cent. |  |  |  |  |  |  |  |
| 1 | 0.63 | 0.63 | 6.93 | 0.44 | 0.137 | 0.44 | R1 | 18 |
| 2 | 0.331 | 0.961 | 3.64 | 0.859 | 0.148 | 0.921 | R1 | 5 |
| 3 | 0.0248 | 0.986 | 0.273 | 0.197 | 0.16 | 0.937 | R1 | 19 |

**Figure S2** Separate PCA for *Rhizoscyphus ericae* (Class 2)

| **Component** | **R2X** | **R2X(cum)** | **Eigenvalue** | **Q2** | **Limit** | **Q2(cum)** | **Significance** | **Iterations** |
| --- | --- | --- | --- | --- | --- | --- | --- | --- |
| 0 | Cent. |  |  |  |  |  |  |  |
| 1 | 0.68 | 0.68 | 6.8 | 0.5 | 0.175 | 0.5 | R1 | 7 |
| 2 | 0.263 | 0.943 | 2.63 | 0.801 | 0.192 | 0.901 | R1 | 7 |
| 3 | 0.0556 | 0.999 | 0.556 | 0.767 | 0.212 | 0.977 | R1 | 4 |
