# Supplementary material for: Investigation of neomycin biodegradation conditions using ericoid mycorrhizal and white rot fungal species
Source: BMC Biotechnol. 2022 Oct 11;22:29. doi: 10.1186/s12896-022-00759-1 (PMC9554996; doi:10.1186/s12896-022-00759-1)
Supplement: Supplementary file 3 [file 12896_2022_759_MOESM1_ESM.docx]

Appendix 1

Table S1 Experimental culture compositions and results

**Experiment**	**Removal degree (%)**	**Removal amount (mg)**	**C_{NS} (g)**	**N_{NS} (g)**	**C_{neo} (g)**	**N_{neo} (g)**	**C_{tot} (g)**	**N_{tot} (g)**	**C_{tot}:N_{tot}**	**C_{neo}:BM**	**C_{tot}:BM**
NS1Tv(1)	2.81	0.29	0.035	0.007	0.005	0.001	0.040	0.0080	5.0	0.128	1.026
NS1Tv(2)	4.57	0.47	0.035	0.007	0.005	0.001	0.040	0.0080	5.0	0.128	1.026
NS2Tv(1)	51.47	5.25	0.144	0.028	0.005	0.001	0.149	0.0293	5.1	0.128	3.821
NS2Tv(2)	50.97	5.20	0.144	0.028	0.005	0.001	0.149	0.0293	5.1	0.128	3.821
NS3Tv(1)	76.54	7.81	0.720	0.140	0.005	0.001	0.725	0.1413	5.1	0.128	18.590
NS3Tv(2)	70.62	7.20	0.720	0.140	0.005	0.001	0.725	0.1413	5.1	0.128	18.590
NS4Tv(1)	61.71	6.29	0.960	0.150	0.005	0.001	0.965	0.1513	6.4	0.128	24.744
NS4Tv(2)	63.10	6.44	0.960	0.150	0.005	0.001	0.965	0.1513	6.4	0.128	24.744
NS4Tv(3)	69.39	7.08	0.960	0.150	0.005	0.001	0.965	0.1513	6.4	0.128	24.744
NS4Re(1)	72.95	7.44	0.960	0.150	0.005	0.001	0.965	0.1513	6.4	0.056	10.722
NS4Re(2)	69.42	7.08	0.960	0.150	0.005	0.001	0.965	0.1513	6.4	0.056	10.722
Neo1Tv(1)	26.26	2.68	0.000	0.000	0.005	0.001	0.005	0.0013	3.8	0.128	0.128
Neo1Tv(2)	24.06	2.45	0.000	0.000	0.005	0.001	0.005	0.0013	3.8	0.128	0.128
Neo1Re(1)	61.69	6.29	0.000	0.000	0.005	0.001	0.005	0.0013	3.8	0.056	0.056
Neo1Re(2)	60.57	6.18	0.000	0.000	0.005	0.001	0.005	0.0013	3.8	0.056	0.056
Neo2Tv(1)	0.00	0.00	0.000	0.000	0.200	0.061	0.200	0.061	3.3	5.128	5.128
Neo2Tv(2)	0.00	0.00	0.000	0.000	0.200	0.061	0.200	0.061	3.3	5.128	5.128
Neo2Re(1)	62.80	188.39	0.000	0.000	0.200	0.061	0.200	0.061	3.3	2.222	2.222
Neo2Re(2)	58.62	175.85	0.000	0.000	0.200	0.061	0.200	0.061	3.3	2.222	2.222
Neo3Tv(1)	0.00	0.00	0.000	0.000	1.210	0.370	1.210	0.37	3.3	31.026	31.026
Neo3Tv(2)	0.00	0.00	0.000	0.000	1.210	0.370	1.210	0.37	3.3	31.026	31.026
Neo3Re(1)	37.25	670.50	0.000	0.000	1.210	0.370	1.210	0.37	3.3	13.444	13.444
Neo3Re(2)	36.50	657.00	0.000	0.000	1.210	0.370	1.210	0.37	3.3	13.444	13.444
Neo4Tv(1)	0.00	0.00	0.000	0.000	2.030	0.620	2.030	0.6200	3.3	52.051	52.051
Neo4Tv(2)	0.00	0.00	0.000	0.000	2.030	0.620	2.030	0.6200	3.3	52.051	52.051
Neo4Re(1)	8.28	372.60	0.000	0.000	2.030	0.620	2.030	0.6200	3.3	22.556	22.556
Neo4Re(2)	7.68	345.60	0.000	0.000	2.030	0.620	2.030	0.6200	3.3	22.556	22.556
