# Supplementary material for: Investigation of neomycin biodegradation conditions using ericoid mycorrhizal and white rot fungal species
Source: BMC Biotechnol. 2022 Oct 11;22:29. doi: 10.1186/s12896-022-00759-1 (PMC9554996; doi:10.1186/s12896-022-00759-1)
Supplement: Supplementary file 4 [file 12896_2022_759_MOESM3_ESM.docx]

Appendix 3

Table S4 Summary of kinetic parameters of degradation of neomycin using different experimental conditions

Experiment	Time (h)	Kinetics	Equation	Half-life (h)	k (mg L^{-1} h^{-1})*	k' (h^{-1})**	Corr. coeff. (r)
NS2Tv (1)	0-168	Ps.-first order	$ln\ C_t = -0.0035t + 3.425$	192.5	n/a	0.0035	0.9666
NS2Tv (2)	0-168	Ps.-first order	$ln\ C_t = -0.0036t + 3.4009$	198.0	n/a	0.0036	0.9759
NS3Tv (1)	0-8	Ps.-first order	$ln\ Ct = -0.0764t + 3.3057$	9.1	n/a	0.0764	0.9467
NS3Tv (2)	0-8	Ps.-first order	$ln\ Ct = -0.0752t + 3.2962$	9.2	n/a	0.0752	0.9473
NS4Tv (1)	0-168	Ps.-first order	$ln\ C_t = -0.0039t + 3.3103$	177.7	n/a	0.0039	0.9362
NS4Tv (2)	0-168	Ps.-first order	$ln\ C_t = -0.0041t + 3.2168$	169.1	n/a	0.0041	0.9593
NS4Tv (3)	0-168	Ps.-first order	$ln\ C_t = -0.0067t + 3.3598$	103.4	n/a	0.0067	0.9759
Neo1Tv (1)	0-8	Ps.-first order	$ln\ C_t = -0.0214t + 3.5208$	32.4	n/a	0.0214	0.9791
Neo1Tv (2)	0-8	Ps.-first order	$ln\ C_t = -0.0240t + 3.5145$	28.8	n/a	0.0240	0.9826
NS4Re (1)	0-8	Zero order	$C_t = -1.4867t + 30.917$	10.4	1.4867	n/a	0.9120
NS4Re (2)	0-8	Zero order	$C_t = -1.6966t + 32.501$	9.6	1.6966	n/a	0.9637
Neo1Re (1)	0-8	Ps.-first order	$ln\ C_t = -0.0570t + 3.4607$	12.2	n/a	0.0570	0.9793
Neo1Re (2)	0-8	Ps.-first order	$ln\ C_t = -0.0458t + 3.4544$	15.1	n/a	0.0458	0.9885

**k*: rate constant for zero order kinetics
***k'*: rate constant for pseudo-first order kinetics
