# Supplementary material for: Investigation of neomycin biodegradation conditions using ericoid mycorrhizal and white rot fungal species
Source: BMC Biotechnol. 2022 Oct 11;22:29. doi: 10.1186/s12896-022-00759-1 (PMC9554996; doi:10.1186/s12896-022-00759-1)
Supplement: Supplementary file 5 [file 12896_2022_759_MOESM5_ESM.docx]

Appendix 5

Table S5 Biomass growth, *R. ericae*

Experiment	Weight BM (mg)	C_{tot} (mg)	C:BM	C_{neo}(mg)	C_{neo}:BM	Removal degree (%)
BMRe (1)	136	35	0.257	0.0	0.0	na
BMRe (2)	141	35	0.248	0.0	0.0	na
NS0Re (1)	157	35	0.223	0.0	0.0	na
NS0Re (2)	184	35	0.190	0.0	0.0	na
NS4Re (1)	141	965	6.844	5.0	0.035	72.95
NS4Re (2)	152	965	6.349	5.0	0.033	69.42
Neo1Re (1)	161	5.0	0.031	5.0	0.031	61.69
Neo1Re (2)	148	5.0	0.034	5.0	0.034	60.57
Neo2Re (1)	185	200	1.081	200	1.081	62.80
Neo2Re (2)	194	200	1.031	200	1.031	58.62
Neo3Re (1)	276	1210	4.384	1210	4.384	48.01
Neo3Re (2)	285	1210	4.246	1210	4.246	47.26
Neo4Re (1)	352	2030	5.767	2030	5.767	8.28
Neo4Re (2)	387	2030	5.245	2030	5.245	7.98

BMRe is the biomass after the 12th day of inoculation
NS0Re is the biomass after 168h growth in NS excluding neomycin

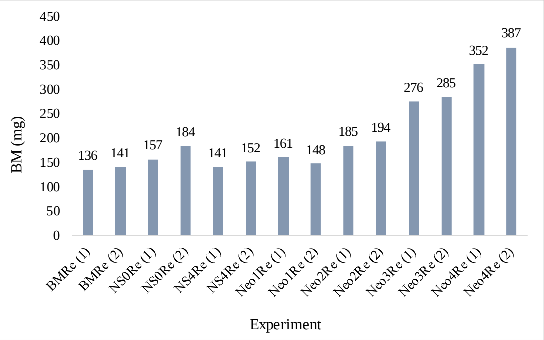

Figure S3 Weight of BM of *R ericae* under different experimental conditions

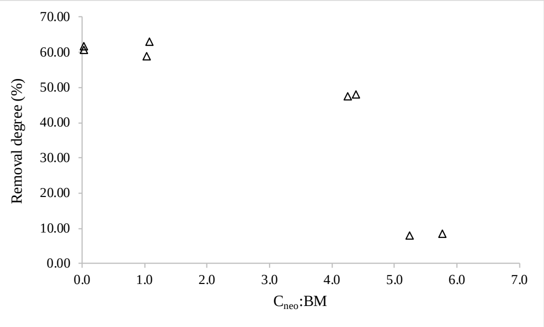

Figure S4 Removal degree using *R. Ericae* as function of C_{neo}/BM ratio
